# Why do intestinal epithelial cells express MHC class II?

**DOI:** 10.1111/imm.13270

**Published:** 2020-10-12

**Authors:** Cornelia Heuberger, Johanna Pott, Kevin Joseph Maloy

**Affiliations:** ^1^ Sir William Dunn School of Pathology University of Oxford Oxford UK; ^2^ Kennedy Institute of Rheumatology University of Oxford Oxford UK; ^3^ Hubrecht Organoid Technology Utrecht Netherlands; ^4^ Institute of Infection, Immunity and Inflammation University of Glasgow Glasgow UK

**Keywords:** CD4+ T cells, gut, intestinal epithelial cell, MHC class II

## Abstract

Intestinal epithelial cells (IECs) constitute the border between the vast antigen load present in the intestinal lumen and the mucosal immune compartment. Their ability to express antigen processing and presentation machinery evokes the question whether IECs function as non‐conventional antigen‐presenting cells. Major histocompatibility complex (MHC) class II expression by non‐haematopoietic cells, such as IECs, is tightly regulated by the class II transactivator (CIITA) and is classically induced by IFN‐γ. As MHC class II expression by IECs is upregulated under inflammatory conditions, it has been proposed to activate effector CD4+ T (Teff) cells. However, other studies have reported contradictory results and instead suggested a suppressive role of antigen presentation by IECs, through regulatory T (Treg)‐cell activation. Recent studies investigating the role of MHC class II + exosomes released by IECs also reported conflicting findings of either immune enhancing or immunosuppressive activities. Moreover, in addition to modulating inflammatory responses, recent findings suggest that MHC class II expression by intestinal stem cells may elicit crosstalk that promotes epithelial renewal. A more complete understanding of the different consequences of IEC MHC class II antigen presentation will guide future efforts to modulate this pathway to selectively invoke protective immunity while maintaining tolerance to beneficial antigens.

## Graphical abstract

Intestinal epithelial cells (IECs) can express MHC class II molecules both at steady state and during inflammation, suggesting that they may shape local CD4+ T‐cell responses towards intestinal antigens. However, the outcome of MHC class II antigen presentation by IECs appears context dependent, resulting in either enhanced pro‐inflammatory responses or in tolerogenic regulatory T‐cell activation. A better understanding of the functions of MHC class II expression by IECs may allow the design of new therapies for intestinal diseases, which either enhance protective immunity or ameliorate harmful inflammation.

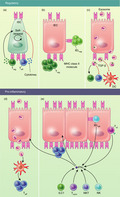

**Abbreviations**
APCs: antigen‐presenting cells
CD: Crohn's disease
cIECs: colonic IECs
CIITA: class II transactivator
CLIP: class II‐associated invariant‐chain peptide
DAMPs: damage‐associated molecular patterns
DCs: dendritic cells
ER: endoplasmic reticulum
GvHD: graft‐vs‐host disease
HA: haemagglutinin
IBD: inflammatory bowel diseases
ICOS‐L: inducible co‐stimulatory ligand
IECs: intestinal epithelial cells
IELs: intraepithelial lymphocytes
Ii: invariant chain
ILC1: innate lymphoid cells 1
ISC: intestinal stem cell
LPLs: lamina propria lymphocytes
MAMPs: microbial‐associated molecular patterns
MHC: major histocompatibility complex class II
MIIC: MHC class II compartment
MLNs: mesenteric lymph nodes
MVB: multi‐vesicular bodies
NK: natural killer cells
NKT: natural killer T cells
OVA: ovalbumin
PD‐L1: programmed death ligand 1
PRRs: pattern recognition receptors
siIECs: small intestinal IECs
siISCs: small intestinal stem cells
TAs: transient‐amplifying cells
Tconv: conventional T cells
TCR: T‐cell receptor
Teff: effector T cell
TGF‐β: transforming growth factor‐β
TLRs: Toll‐like receptors
Treg: regulatory T cell
UC: ulcerative colitis

## INTRODUCTION

The human gastrointestinal tract has a surface area of around 30 m^2^,^1^ and its lumen is colonized by more than 10^13^ bacteria.^2^ The intestinal epithelium has two main functions: absorption of nutrients and water and providing a physical and chemical barrier against the luminal microbiota. Intestinal epithelial cells (IECs) execute important functions to help regulate immune homeostasis. Loss of epithelial barrier integrity can result in the translocation of luminal components and may contribute to the breakdown of intestinal homeostasis observed in certain intestinal inflammatory disorders, such as inflammatory bowel diseases (IBD).^3^ Although IECs were shown to express MHC class II several decades ago, the functional importance remains unclear. In this review, we explore the expression of the antigen processing and presentation machinery by IECs. We discuss the possible interactions of MHC class II + IECs with CD4+ T cells and the potential functional impacts on immunity and inflammation, as well as crosstalk to facilitate intestinal stem cell (ISC) renewal. We also discuss how release of MHC class II‐bearing vesicles by IECs may modulate immune responses.

## THE INTESTINAL EPITHELIUM ARCHITECTURE

**Figure 1:**
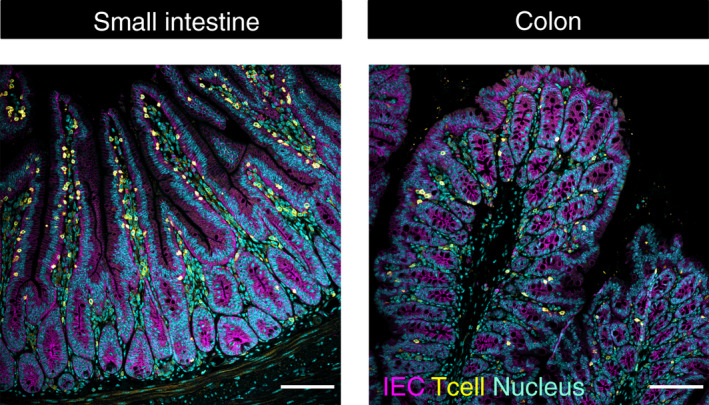
The intestinal epithelial architecture. Formalin‐fixed, paraffin‐embedded sections of the small intestine (left panel) and colon (right panel) from a C57Bl/6 mouse were immunofluorescently stained as described elsewhere.^93^ Β‐catenin (marking intestinal epithelial cells (IECs)) is shown in magenta, CD3 (marking T cells) in yellow and nuclei in cyan. The small intestine is organized in crypt–villus units whereas the colon lacks villi and only consists of crypts, which open into a flat epithelium. The intestinal epithelium is structurally polarized, with the apical surface facing the intestinal lumen and the basolateral membrane the lamina propria. T cells are located in close contact to the intestinal epithelium. They can be found in between the IECs or in the lamina propria both in the small intestine and in the colon; however, T‐cell numbers are higher in the small intestine compared to the colon. Scale bar depicts 100 μm

The intestinal epithelium is composed of a single layer of columnar IECs. It facilitates the absorption of metabolites and water while compartmentalizing the immune cells residing in the lamina propria from the microbiota in the lumen. Its large surface is formed by millions of crypt–villus units.^4^ Each villus, a finger‐like structure extending into the lumen, is surrounded by multiple crypts. In the small intestine, the villi are composed of post‐mitotic IECs and their length decreases along the gastrointestinal tract. In the colon, villi are lacking and the crypts open into a flat epithelium (Figure 1).

To maintain the epithelial barrier in the face of constitutive exposure to a vast microbiota and mechanical stress caused by peristaltic movement, it is constantly replenished by ISCs. ISCs reside at the bottom of the crypt and give rise to progenitor cells, called transient‐amplifying cells (TAs) that proliferate and differentiate into mature IECs. The epithelium consists of at least six different mature cell types, which can be separated into absorptive (enterocytes and M cells) and secretory (Paneth, goblet, enteroendocrine and tuft cells) cells.^5^, ^6^

The intestinal epithelium plays a vital part in maintaining immune homeostasis with the commensal microbiota, partly because it limits the physical interaction of the microbiota with the immune cells residing in the lamina propria. This physical barrier is enforced by inducible defence mechanisms; recognition of the microbiota by IECs can trigger their activation and expression of mediators, such as chemokines or cytokines, which subsequently can activate local immune cells.^7^, ^8^ These defences are activated by pattern recognition receptors (PRRs), which recognize microbial‐associated molecular patterns (MAMPs) or damage‐associated molecular patterns (DAMPs) released from the host cells. IECs express a variety of different PRRs, and signalling through Toll‐like receptors (TLRs) was shown to maintain barrier function.^7^, ^8^ IECs are structurally polarized – their apical surface faces the intestinal lumen, whereas their basolateral membrane is in contact with the lamina propria – and TLR activation can lead to different cellular responses depending on the localization at either the apical or basolateral membrane.^9^, ^10^, ^11^ Additionally, TLR expression levels by IECs change along the intestine with small intestinal IECs (siIECs) generally expressing lower levels than colonic IECs (cIECs),^10^ which corresponds to the much lower levels of microbiota found in the small intestine. Specialized IEC populations may also detect and direct responses to distinct pathogens; for example, tuft cells have recently been demonstrated to participate in immunity against some parasitic worms through release of IL‐25 that promotes type II responses.^5^, ^12^, ^13^, ^14^

Although it is clear that IECs make crucial contributions to barrier integrity and innate immune pathways, evidence of their participation in antigen‐specific immune reactions is more equivocal. Nevertheless, their ability to express major histocompatibility complex (MHC) class II proteins, as well as associated processing molecules, suggests that IECs may function as non‐conventional antigen‐presenting cells (APCs).

## THE MHC CLASS II PROCESSING AND PRESENTATION PATHWAY

The MHC class II antigen processing and presentation pathway are a highly regulated process which involves many accessory proteins. As the newly synthesized MHC class II molecules are located in the endoplasmic reticulum (ER), the premature binding of peptides in the ER needs to be prevented. Therefore, the MHC class II molecules assemble with the MHC class II invariant chain (Ii or CD74), part of which occupies the peptide‐binding groove of the MHC class II molecule, thus preventing the binding of other peptides.^15^, ^16^ The complex travels through the endosomal pathway where Ii gets progressively trimmed until MHC class II only contains the short class II‐associated invariant‐chain peptide (CLIP).^17^, ^18^ The cleavage of Ii to CLIP is thought to occur in the acidic MHC class II compartment (MIIC) late in the endosomal pathway. Within MIIC, binding of the MHC class II‐like molecule HLA‐DM to the MHC class II molecule: CLIP complex catalyses the release of CLIP from the peptide‐binding groove and stabilizes the empty MHC class II molecule to allow the binding of antigenic peptides.^19^, ^20^, ^21^ Endocytosed proteins from extracellular origin as well as self‐proteins are initially proteolysed in endosomal and lysosomal antigen processing compartments, which generates peptide fragments. In addition to antigenic peptides from extracellular origin, MHC class II molecules can also cross‐present cytosolic self‐antigen and foreign antigen processed by autophagy. This naturally occurring process directs damaged organelles and cytosolic proteins into autophagosomes during stress or starvation. The cargo is subsequently transported via the endosomal pathway into lysosomes where they are degraded and the resultant peptides may encounter MHC class II molecules embedded in the membrane.^22^, ^23^, ^24^ Thus, MHC class II molecules can present a highly diverse repertoire of peptides which originate from a range of cellular compartments of APCs.^22^, ^25^, ^26^ After the MHC class II containing vesicle fuses with the antigenic peptide containing compartment, the peptides can bind to MHC class II molecules on their way to the cell surface. The stable MHC class II:peptide surface complexes can be recognized by cognate antigen‐specific CD4+ T cells.

## MHC CLASS II EXPRESSION AND LOCALIZATION IN IECS

Several decades ago, it was shown that IECs were able to express MHC class II molecules, thereby possessing an important prerequisite to function as non‐conventional APCs.^27^, ^28^, ^29^, ^30^ Human siIECs constitutively express MHC class II molecules^28^, ^30^, ^31^, ^32^, ^33^ and it was noted that this was most evident at the tip of the villus. However, it was recently reported that murine ISC subsets at the base of the small intestinal crypts also express MHC class II under homeostatic conditions.^34^ In contrast, cIECs do not express MHC class II molecules under healthy, steady‐state conditions. However, MHC class II is upregulated both in human and in murine small intestinal and colonic IECs under inflammatory conditions, including the immune‐mediated disorders IBD, coeliac disease and graft‐vs‐host disease (GvHD), as well as during infections with *Salmonella enterica* or *Heligmosomoides polygyrus* ^31^, ^34^, ^35^, ^36^, ^37^, ^38^ (Table 1). Hershberg et al^39^ demonstrated that in the presence of the pro‐inflammatory cytokine IFN‐γ, human colonic IEC‐like cell lines express MHC class II on their cell surface as well as Ii and HLA‐DM. They also showed that, without additional activation, these human colonic IEC‐like cell lines were able to process and present antigen from the apical surface of cIECs, but only when high concentrations of antigen were present.^39^ Moreover, when the human colonic IEC‐like cell line was transfected with class II transactivator (CIITA), mimicking pro‐inflammatory conditions, the IECs were able to take up and process antigen from both the basolateral and apical membrane.^40^

**Table 1:** MHC class II expression by intestinal epithelial cells

	Small intestine	Colon	Species
Homeostasis			
Steady state^28^, ^30^, ^31^, ^32^, ^33^, ^37^	+	−	Human, Mouse
Germ free^37^, ^45^	−	N.d.	Mouse
MyD88−/− Trif−/−^37^	−	−	Mouse
Rag−/− IL2rg−/−^37^	−	N.d.	Mouse
Inflammation/Infection			
Coeliac disease^31^, ^47^, ^48^, ^49^	++	N.d.	Human
Inflammatory bowel disease ^35^, ^36^, ^38^	++	++	Human
Graft‐versus‐host disease^37^	++	N.d.	Mouse
*Salmonella enterica* ^34^	++ (siISC)	N.d.	Mouse
*Heligmosomoides polygyrus* ^34^	++(siISC)	N.d.	Mouse

Note

Various factors contribute to the expression of MHC II molecules by IECs under different conditions. During homeostasis, small intestinal IECs (siIECs) express MHC class II, whereas colonic IECs (cIECs) do not. Homeostatic MHC class II expression by siIECs was described to be dependent on the microbiota as well as Toll‐like‐receptor signalling via MyD88/TRIF and the presence of innate and adaptive lymphocytes. During inflammatory conditions, MHC class II expression was upregulated in IECs and intestinal infections led to an increase in MHC class II expression in small intestinal stem cells (siISCs). +, MHC class II is expressed. −, MHC class II is not expressed. ++, MHC class II expression is upregulated. N.d., not determined.

In terms of localization of MHC class II molecules in IECs, using a human colonic cell line transfected with a MHC class II construct, MHC class II was shown to be predominantly located at the basolateral membrane.^40^ However, analyses of human and rat siIECs showed MHC class II molecules located at basolateral, lateral and apical membranes as well as in intracellular vesicular structures.^33^, ^35^, ^41^ The uptake of antigen is mainly mediated by the endosomal pathway, where early endosomes progressively develop into the late endosomal structure MIIC. The latter can be divided into distinct compartments according to ultrastructural morphology, which includes multi‐vesicular bodies (MVB).^42^ Several studies characterized the localization of MHC class II molecules within IECs in endoscopic biopsies of healthy controls and IBD patients^33^, ^43^ as well as human small intestinal organoids.^44^ In the healthy human gut, the late endosomal structure MVB harboured the majority of MHC class II molecules, but they were also observed at the basolateral cell membrane with faint expression at the apical membrane.^33^, ^43^ The localization of MHC class II in late endosomal structures was also detected in human siIEC organoids.^44^ Bär et al showed that in inflamed samples from Crohn's disease (CD) and ulcerative colitis (UC) patients, the expression of MHC class II molecules increased at the basolateral membrane, which was accompanied with a decrease of MHC class II molecules located in MVB.^43^ These observations support the hypothesis that MHC class II molecules get transported from MVB to the basolateral membrane during inflammation and that this redistribution potentially allows IEC to present antigen to CD4+ T cells in the lamina propria during such conditions.

## INDUCTION OF MHC CLASS II EXPRESSION BY IECS

The dichotomy between constitutive MHC class II expression in the small but not in the large intestine and the conserved upregulation in both sites during inflammatory conditions suggests that distinct environmental signals may induce MHC class II expression by IECs.

During homeostasis, IL‐12 (mainly produced by macrophages) induces the secretion of IFN‐γ by lamina propria lymphocytes (LPLs) in the small intestine.^37^ A role for the microbiota in the induction of MHC class II expression by siIECs is supported by observations that antibiotic treatment of mice reduced both IL‐12 levels and MHC class II expression by siIECs and by the lack of epithelial MHC class II expression in germ‐free mice.^37^, ^45^ Consistent with a requirement for microbial sensing, siIECs of MyD88−/− TRIF−/− mice do not express MHC class II at steady state or during inflammation.^37^ However, whether IFN‐γ is the sole inducer of MHC class II expression on IECs is not entirely clear, particularly as MHC class II molecules were detected on siIECs in mice lacking IFN‐γR, highlighting potential IFN‐γ independent induction mechanisms.^46^ However, expression of Ii and CIITA mRNA was low in IFN‐γR‐deficient IEC,^46^ which might suggest reduced antigen presentation capabilities. In addition, a subsequent study reported that IFN‐γR−/− mice did not express MHC class II on IECs, which supports the importance of IFN‐γ as a critical inducer.^37^ Different environmental factors of the IFN‐γR−/− mice in the different studies, such as diet and microbiota, might have contributed to these conflicting results. Indeed, the food antigen gliadin was shown to increase MHC class II expression by siIECs in coeliac disease^47^, ^48^ and withdrawal of the antigen resulted in reduced siIEC MHC class II levels.^31^, ^49^ However, the effects of gliadin are likely to have been indirect, as it can drive activation of IFN‐γ‐secreting T cells that drive inflammation.^50^ Cells which potentially release IFN‐γ in the intestine include innate lymphoid cells 1 (ILC1),^51^ natural killer cells (NK),^37^ natural killer T cells (NKT),^37^ γδ T cells,^52^ as well as conventional T cells (CD4+ and CD8+ T cells).^37^ During both homeostasis and inflammation, mice lacking adaptive and innate lymphocyte populations (Rag2−/− IL2rg−/−) completely lacked MHC class II expression on siIECs, whereas those lacking conventional T cells only (Rag2−/−) showed reduced levels of MHC class II on siIECs.^37^ Thus, it appears that both innate and adaptive lymphocytes contribute to IFN‐γ‐dependent induction of MHC class II on siIECs at steady state (Table 1).

The development of mouse and human intestinal organoid cultures, which form three‐dimensional structures that resemble the intestinal architecture, has facilitated analysis of primary IEC responses to defined stimuli.^53^ The loss of MHC class II expression by siIEC organoids in prolonged ex vivo cultures suggests extrinsic stimulation is critical for continued expression of MHC class II.^34^, ^37^ Basolateral IFN‐γ stimulation induced MHC class II expression by human siIEC organoids^37^, ^44^ and murine small intestinal organoids.^37^, ^54^

MHC class II expression is transcriptionally controlled by CIITA. In non‐haematopoietic cells, including IECs, CIITA expression is regulated by the pIV promoter, which is activated by IFN‐γ.^55^ Although IFN‐γ is clearly a key regulator of MHC class II expression in IECs and contributes to the increase observed during intestinal inflammation, there may be other signals that can induce MHC class II expression on IECs. For example, human IEC cell lines express the IL‐27 receptor complex and IL‐27 was found to induce CIITA expression in IECs.^56^ Although it was not reported whether this led to increased MHC class II expression on IECs,^56^ it is noteworthy that IL‐27 induced increased expression of MHC class II molecules on HUVEC cells.^57^ Whether suppressors of IFN‐γ‐mediated MHC class II expression exist in IECs has still to be determined. In various other non‐haematopoietic cell types, an inhibitory capacity via the pIV promoter of CIITA has been linked to transforming growth factor‐β (TGF‐β),^58^ IL‐1β^59^ as well as the pathogens cytomegalovirus^60^, ^61^ and chlamydia.^62^ In addition, IL‐4 and IL‐10 were shown to inhibit IFN‐γ induced CIITA expression in mouse microglia cells but the molecular mechanism remains unclear.^63^

In summary, the microbiota appears to indirectly drive MHC class II expression by IECs by activating an IL‐12‐IFN‐γ circuit in local leucocytes. However, whether additional cytokines can induce MHC class II expression by IECs, and whether pathogens and regulatory factors can downregulate their MHC class II expression remain to be established.

## IECS SHOW LIMITED EXPRESSION OF CO‐STIMULATORY MOLECULES

After the binding of the TCR to the MHC class II:peptide complex, co‐stimulatory molecules co‐localize to the complex. Co‐stimulatory signal transduction shapes the flavour of the T‐cell response. Depending on the ligand, it can either promote or inhibit T‐cell activation, differentiation and function. Co‐stimulatory molecules include CD80, CD86, CD40 and inducible co‐stimulatory ligand (ICOS‐L). Conversely, programmed death ligand 1 (PD‐L1) is a co‐inhibitory molecule that transduces an inhibitory signal into T cells.^64^

Conflicting results have been generated whether IECs are capable of delivering co‐stimulatory or co‐inhibitory signals. The co‐stimulatory molecules CD80, CD86 and CD40 were reported to be expressed at the mRNA level in healthy human IECs.^32^, ^65^ In contrast, another study reported a lack of CD80/CD86 mRNA expression in murine siIECs, even after IFN‐γ treatment.^66^ Moreover, the detection of these molecules at the protein level appears to be driven by inflammation in the tissue. Similarly, while some studies reported an absence of CD80, CD86 and CD40 protein expression in healthy human IECs^32^, ^67^ and CD80/CD86 protein expression in cIECs in UC patients,^68^ others reported CD86 protein expression by cIECs in UC patients^65^ and CD40 protein expression was detected in inflamed colonic and ileal IECs of CD and UC patients, as well as in IEC‐like cell lines after IFN‐γ stimulation.^67^ In addition, CD80 protein levels were upregulated on siIECs during GvHD.^37^

Although mRNA for both the co‐stimulatory molecule ICOS‐L and the co‐inhibitory molecule PD‐L1 were detected in healthy and inflamed cIECs of IBD patients, only low surface expression of ICOS‐L and PD‐L1 was detected in patient cIECs, with PD‐L1 being more highly expressed by cIECs in CD than in UC patients.^69^

Taken together, mRNA for different co‐stimulatory and co‐inhibitory molecules appears to be expressed by IECs both at steady‐state and under inflammatory conditions. Although the detection of surface expression of co‐stimulatory and co‐inhibitory molecules was not consistent across the different studies, when detected these molecules were expressed at low levels and were mainly present in inflamed tissue. Therefore, there is no clear evidence that IECs can provide sufficient co‐stimulatory signals to activate naïve CD4+ T cells. However, as noted below, intestinal T‐cell populations mainly comprise activated effector and regulatory T cells that are less dependent on co‐stimulatory signals for triggering of their effector functions.^70^ Thus, it seems likely that IECs modulate Teff and Treg cell responses to maintain intestinal homeostasis.

## THE INTESTINE HARBOURS PREDOMINANTLY ACTIVATED T CELLS

Conventional APCs such as dendritic cells (DCs), B cells, macrophages, as well as thymic epithelial cells, all express MHC class II molecules. They activate naïve CD4+ T cells through the binding of the T‐cell receptor (TCR) and co‐receptor CD4 to the MHC class II:peptide complex. For full activation, naïve CD4+ T cells additionally need co‐stimulatory molecule signalling and differentiation into distinct effector CD4+ T (Teff) cell subsets is directed by the surrounding cytokine milieu.^71^ CD4+ T cells can be functionally divided into Teff subsets, such as Th1, Th2 and Th17 cells, and the suppressive regulatory T (Treg) cells.^71^

The intestinal CD4+ T cells are located in two distinct compartments: the lamina propria and the intestinal epithelium (Figure 1). Naïve CD4+ T cells circulate in the blood stream until they enter the GALTs in the intestine or the draining mesenteric lymph nodes (MLNs), a tightly regulated process involving distinct chemokines and chemokine receptors.^72^, ^73^ In these lymphoid tissues, migratory DCs present antigens which they have previously taken up at mucosal sites, as well as co‐stimulatory molecules and cytokines, to naïve CD4+ T cells.^74^ The primed antigen‐specific T cells differentiate into CD4+ Teff and Treg cells. The induction of Treg cells is considered to be dependent on CD103+ DCs and their secretion of soluble factors such as retinoic acid and TGF‐β.^75^ Primed T cells migrate via the bloodstream and home to the intestinal lamina propria – the gut‐specific homing process being orchestrated by adhesion molecules, chemokines and chemokine receptors.^76^ Thus, the lamina propria harbours mainly antigen‐experienced Teff and Treg cells, with very few naïve CD4+ T cells. Antigen presentation by IECs might therefore be primarily important for modulation and maintenance of these previously primed T cells, rather than in the activation of naïve T cells.

## T‐CELL MODULATION BY ANTIGEN PRESENTATION BY IECS

**Figure 2:**
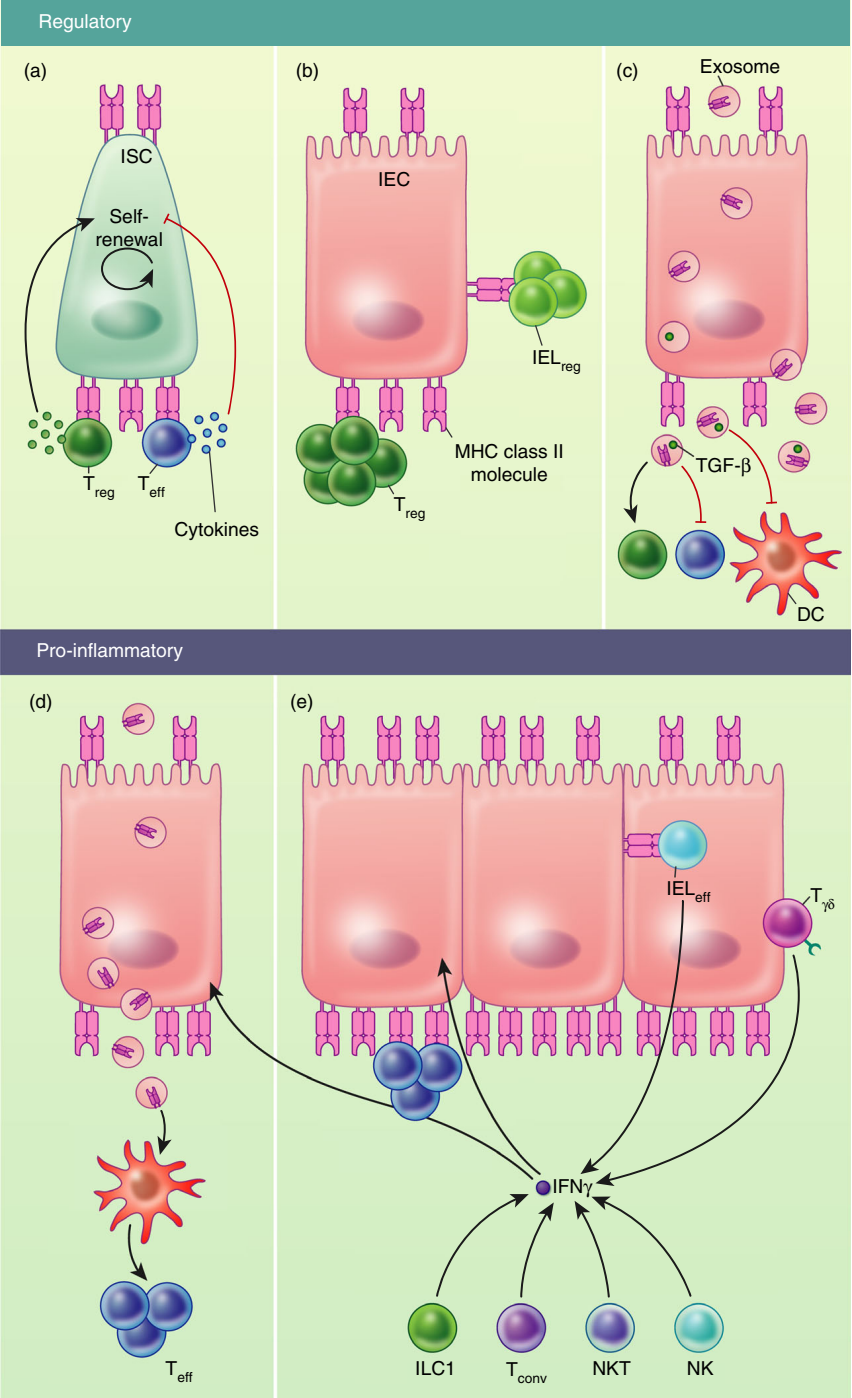
Potential effects of MHC class II expression by IECs on regulatory and pro‐inflammatory circuits in the gut. Different studies have proposed a variety of potential mechanisms through which MHC class II expression by IECs may influence local immune activity. (A) MHC class II expression by small intestinal stem cells (siISCs) was described to play a role in the crosstalk with lamina propria CD4+ T cells,^34^ with cytokines released by regulatory T (Treg) cells or effector T (Teff) cells promoting siISC stem cell renewal and differentiation, respectively.^34^ (B) Intestinal epithelial cells (IECs) expressing MHC class II were shown to boost Treg cell expansion both in the lamina propria^84^ and in the intestinal epithelium.^83^ (C and D) Moreover, IECs may indirectly modulate adaptive immune responses via the release of MHC class II + exosomes, although whether IEC exosomes induce a tolerogenic^90^, ^91^ (C) or pro‐inflammatory^89^, ^92^ (D) immune response is unclear. (E) During inflammation, the pro‐inflammatory cytokine IFN‐γ gets potentially released by innate lymphoid cells 1 (ILC1),^51^ natural killer cells (NK),^37^ natural killer T cells (NKT),^37^ γδ T cells (Tγδ),^52^ as well as conventional T cells (Tconv) (CD4+ and CD8+ T cells)^37^ in the intestine. IFN‐γ was shown to upregulate MHC class II expression by IECs,^37^, ^79^ which can enhance Teff cell functions in the lamina propria^36^, ^37^ as well as intestinal epithelial lympocyte (IEL) functions in the epithelium.^79^ IELs released IFN‐γ upon interaction with epithelial MHC class II,^79^ which in turn might sustain MHC class II expression by IECs. DC, dendritic cell

Their close proximity to T lymphocytes within the epithelium and the lamina propria, allied to their ability to take up, process and present antigen, makes it plausible that IECs modulate local antigen‐specific CD4+ T cells. However, the functional outcomes of such interactions are the subject of ongoing debate, as it is not clear whether IECs primarily enhance Teff cell activity, or whether they render T cells anergic or tolerant (Figure 2).

### **Effector CD4**+ **T‐cell activation by IECs**

Co‐culture of primary IECs ex vivo with T cells is challenging, as isolated individual IECs show poor survival upon dissociation from the mucosa. An additional drawback is the potential contamination of the IEC suspension with other cells during the isolation process, particularly conventional APCs that express high levels of MHC class II and co‐stimulatory molecules.^34^ Therefore, most previous in vitro work was carried out with IEC‐like cell lines – mainly derived from carcinoma cells. Moreover, some of these colorectal cancer cell lines were shown to be susceptible to epigenetic changes which can silence CIITA gene expression.^77^

Nevertheless, several studies have reported the in vitro activation of T‐cell lines and hybridomas, which are more akin to activated effector T cells than to naïve T cells, by MHC class II antigen presentation by IEC‐like cell lines.^39^, ^40^, ^78^ Furthermore, Dotan et al reported that co‐culture of primary cIECs from IBD patients with autologous or allogeneic healthy peripheral blood T cells induced the proliferation of CD4+ T cells and the release of IFN‐γ, which was inhibited by blockade of MHC class II.^36^ In addition, Hatano et al reported that co‐culture with IFN‐γ‐pretreated murine siIECs stimulated IFN‐γ secretion by CD4+ intraepithelial lymphocytes (IELs), but not by LPLs or primed splenic T cells.^79^ These findings support the potential for activation of T cells by IECs, but also highlight potential differences depending on the tissue and species origin of the T cells and IECs.

A recent study identified a key role for MHC class II expression by siIECs during experimental GvHD.^37^ This disease is driven by mature donor T cells which recognize host alloantigens and leads to severe inflammation in several tissues, especially the small intestine.^80^ Using conditional gene ablation of MHC class II expression in IECs, they found that siIECs activated donor CD4+ T cells, which caused GvHD‐induced lethality in vivo.^37^ This GvHD also involved the intestinal microbiota and was driven by the IL‐12/IFN‐γ cytokine axis, as therapeutic targeting of this pathway led to decreased lethality.^37^ This study demonstrated that MHC class II antigen presentation by siIECs plays a critical role in GvHD‐driven intestinal pathology in vivo.

These findings in GvHD contrast with a previous report that also assessed the contribution of MHC II antigen presentation by IECs during intestinal inflammation. They generated transgenic mice in which MHC class II expression was restricted to either IECs (fatty acid binding protein promoter) or DCs (CD11c‐promoter) and found that MHC class II antigen presentation by CD11c+ cells (DCs) only was sufficient to elicit severe intestinal pathology following adoptive transfer of CD4+ T cells into Rag2−/− recipients.^81^ In contrast, colitis was not induced when MHC class II expression was restricted to IECs in the Rag2−/− recipients.^81^ However, whether MHC class II expression by IECs was insufficient to induce T‐cell transfer colitis due to their inability to activate naïve CD4+ T cells and/or due to the preferential induction of Treg cells or anergy was not determined. The seemingly disparate conclusions of these studies on the ability of MHC class II antigen presentation by IEC to drive intestinal inflammation likely reflect distinct contextual features of the experimental models. GvHD represents small intestinal pathology induced by an unusually high frequency of alloreactive T cells in intestinal tissue that has suffered significant damage from the experimental procedure (irradiation). In contrast, the T‐cell transfer model of colitis assesses chronic colonic inflammation that arises after priming of microbiota‐specific naïve CD4+ T cells in the absence of a Treg cell compartment. Thus, the responding T cells as well as the local microenvironment are significantly different, but both studies are consistent with the viewpoint that MHC II expression by IEC primarily acts to influence antigen‐experienced Teff cells and not naïve T cells.

### **Regulatory CD4**+ **T‐cell activation by IECs**

The general lack of co‐stimulatory molecule expression and reported expression of co‐inhibitory molecules by IECs led to the opposing hypothesis that they could preferentially induce immunosuppressive Treg cell responses. Indeed, several studies have suggested such a regulatory function of MHC class II presentation by IECs.

For example, Westendorf et al^82^ showed that the expression of autoantigen by IECs led to the expansion of antigen‐specific CD4+ FoxP3+ Treg cells in the intestinal mucosa. In the same vein, a recent study investigated transgenic mice that lacked MHC class II expression in all non‐haematopoietic cells (including IECs) and found that these mice developed more severe chronic T‐cell‐mediated colitis than wild‐type littermates.^83^ The exacerbated inflammation was associated with increased expression of the Th1‐associated transcription factor T‐bet in the colon and reduced proportions of Treg cells in the intestinal epithelium.^83^ MHC class II expression by cIECs of colitic mice was mainly induced by IFN‐γ secreted by CD4+ T cells, and they did not detect the co‐stimulatory molecules CD40, CD80 and CD86 on the surface of cIECs.^83^ This suggests that the local release of IFN‐γ by immune cells induces MHC class II expression by IECs, which may temper inflammation by favouring activation of Treg cells.

Westendorf et al^84^ subsequently showed that constitutive expression of influenza haemagglutinin (HA) by siIECs (IEC‐HA) leads to the expansion of HA‐specific peripheral Treg cells in the lamina propria and MLNs as well as the induction of HA‐specific CD4+ T‐cell proliferation. Furthermore, they found that adoptive transfer of these antigen‐experienced Treg cells from IEC‐HA transgenic mice prevented the induction of diabetes in mice expressing HA under the control of the rat insulin promoter that also received HA‐specific naïve CD4+ T cells.^84^ Treg cell expansion by IECs was independent of DCs, TGF‐β and retinoic acid. In addition, IEC stimulated CD4+ T‐cell proliferation, comprising both Treg and Teff cells, was shown to be dependent on MHC class II interaction.^84^ Suppressive effects of IECs on CD4+ T‐cell activation were also reported in another independent study.^85^ They found that co‐culturing of ovalbumin (OVA) pulsed cIECs with OVA‐specific naïve or antigen primed CD4+ T cells inhibited their activation, through a mechanism that appeared to be cell contact dependent, as cIEC conditioned media did not mediate CD4+ T‐cell suppression.^85^ However, whether the inhibition was mediated by MHC class II antigen presentation was not examined. Furthermore, the responsiveness of CD4+ T cells previously co‐cultured with cIECs was restored when these CD4+ T cells were re‐challenged in a co‐culture with conventional APCs,^85^ suggesting that cIECs transiently suppressed CD4+ T‐cell activation, rather than rendering them permanently unresponsive.

The conflicting observations regarding the ability of IECs to stimulate effector or regulatory T‐cell responses highlight the need for further investigations into the role of antigen presentation by IECs, both at steady‐state and under inflammatory conditions. To directly assess the interactions of IECs with T cells future work could perform co‐culture assays with ex vivo cultured IEC organoids. These organoid cultures can be established from both murine and human primary IECs. They should provide a simplified model, which allows the addition of different T‐cell subsets as well as cytokines and antigens/peptides to study the MHC class II‐dependent interactions of IECs with T cells. Previous work illustrated uptake of apoptotic IECs by DCs, which may be important for the induction and maintenance of self‐tolerance;^86^ however, more recent findings suggest that DC‐independent Treg cell expansion can be induced by IECs.^84^ The full extent of the interactions between intestinal DCs and IECs involved in the induction and maintenance of self‐tolerance towards intestinal antigens remains to be determined.

## IECS MAY INDIRECTLY MODULATE ADAPTIVE IMMUNE RESPONSES VIA EXOSOMES

Several studies have provided evidence that MHC class II molecules expressed by IECs may modulate T‐cell responses by indirect mechanisms. Intercellular communication can be mediated by exosomes, which are released extracellularly after the fusion of multi‐vesicular endosomes with the cell membrane.^87^ IEC‐like cell lines were shown to secrete exosomes carrying increased MHC class II molecules after IFN‐γ treatment.^88^, ^89^ In an ovalbumin oral tolerance model, intraperitoneal injection of siIEC‐like cell line‐derived exosomes containing MHC class II molecules as well as ovalbumin did not result in a tolerogenic response, but instead led to increased levels of OVA‐specific IgE and IgG antibodies.^89^ This is in contrast to an earlier study that described the induction of tolerance by intravenous injection of rats with serum‐containing MHC class II+ exosomes that were obtained from rats fed an OVA containing diet.^90^ They further showed that depletion of MHC class II + exosomes using anti‐MHC class II antibodies rendered the serum incapable of transferring tolerance.^90^ Similarly, Jiang et al^91^ showed that IECs produced MHC class II + exosomes containing high levels of TGF‐β that suppressed the proliferation of CD4+ T cells in vitro – a process mediated by TGF‐β. In addition, the intravenous transfer of IEC‐derived exosomes into mice reduced the pathology of DSS induced colitis, partially through the development of Treg cells and inhibition of DC activation.^91^ However, one key caveat of the studies described above is that whether these effects are functionally dependent on MHC class II molecules present in such exosomes remains uncertain. Indeed, another study observed the uptake of cIEC‐like cell line‐derived MHC class II + exosomes by DCs^92^ and showed that the DCs acquired peptides presented by the MHC class II:peptide complexes and lower exosome‐derived peptide doses were needed for DCs to subsequently activate T‐cell hybridomas compared to DCs pretreated with soluble peptide.^92^

These studies suggest that MHC class II+ exosomes released by IECs could transfer antigen to local APCs, thereby initiating and/or shaping adaptive immune responses. It is, however, not clear whether DCs internalize the exosomes and present the IEC‐derived MHC class II:peptide complexes or whether the exosomes act as a source of intact antigen that is then degraded and presented on newly synthesized MHC class II molecules in the DC. The co‐culture of MHC class II‐deficient DCs with ex vivo intestinal organoids could shed light on this question, as the uptake and presentation of IEC‐derived MHC class II:peptide complexes would be possible whereas re‐processing and presentation would not.

## A POTENTIAL ROLE OF MHC CLASS II EXPRESSION IN ISC HOMEOSTASIS

Previous work has focused on the T‐cell response following an antigen‐dependent interaction with IECs. However, the recognition of epithelial MHC class II:peptide complexes by T cells might also facilitate the crosstalk of T cells with the IECs themselves.

Using single‐cell RNA seq analysis of murine siIECs, Biton et al described high expression of MHC class II molecules on two distinct ISC subsets, both of which were highly proliferative.^34^ Those ISC subsets were able to take up and process extracellular antigen and co‐culture of sort purified, peptide‐pulsed siISCs with naïve antigen‐specific T cells stimulated IL‐2 release and T‐cell proliferation, which were absent if ISCs lacked MHC class II molecules.^34^ In addition, co‐culture of primary siIEC organoids with different polarized, cytokine producing Th cell subsets, or their respective cytokines, influenced the differentiation of IECs: Treg co‐culture led to the expansion of ISCs while Th1, Th2 or Th17 co‐culture led to the differentiation of IECs into TA cells.^34^ Evidence supporting in vivo crosstalk between siISCs and CD4^+^ T cells was provided by observations that mice with conditional knock out of MHC class II expression in IECs, or those lacking T cells, exhibited increased frequencies of siISCs. Conversely, depletion of Treg cells led to a reduction in ISCs, which might be augmented by the accompanying increase in Th cell subsets.^34^ Taken together, these findings suggest that ISCs can express MHC class II molecules and interact with diverse CD4+ T cells in an antigen‐dependent manner. The resultant crosstalk may be important in modulating appropriate epithelial renewal and differentiation during episodes of inflammation and infection.

## CONCLUSION AND PERSPECTIVES

The studies discussed in this review highlight that MHC class II expression by IECs can have diverse functional outcomes depending on the IECs themselves, the interacting T cells and the immunological status of the tissue (Figure 2). However, there are many areas requiring further study, to better understand how the site and context of MHC class II expression by IECs shapes local immune homeostasis. These include identification of the signalling pathways which drive constitutive expression of MHC class II in the small intestine at steady state, as well as the homeostatic functions of IEC MHC class II antigen presentation in this site. The antigens processed and presented by IECs during different conditions in vivo also remain largely unknown and could provide important clues as to whether MHC class II antigen presentation is geared towards boosting anti‐pathogen inflammatory T cells or to promoting tissue‐reparative processes. The relative importance of direct MHC class II antigen presentation by IECs vs. indirect modulation of T‐cell responses via local APC also remains unclear. Furthermore, the complex interactions of the microbiota, conventional APCs and T‐cell responses in regulating MHC class II expression by IECs are challenging to untangle. Advances in ex vivo IEC organoid cultures, together with the capacity for single‐cell gene expression analysis, should facilitate large‐scale interrogation of T‐cell–IEC interactions under defined conditions, which in turn should identify key pathways that are regulated by such interactions in both cell types. Validation of these circuits will require analysis of relevant models of intestinal infection and/or inflammation as well as profiling of stratified patient tissue samples. Together, such studies should provide high level insight into the functional relevance of MHC class II antigen presentation by IECs during different circumstances and may provide a rationale for selective modulation of this pathway to enhance protective immunity or to ameliorate harmful inflammation.

## CONFLICT OF INTEREST

The authors have no competing interests to declare.

## ACKNOWLEDGMENTS

K.J.M and J.P. were funded by the MRC Project Grant MR/N02379X/1. K.J.M. is supported by a Wellcome Trust Investigator award (102972). C.H. was supported by a Graduate Student Scholarship from the MRC (MR/R502224/1).

## Data Availability

Data sharing is not applicable to this article as no new data were created or analysed in this study.
